# 724 How Nursing Turnover Rates Effected a Series of Pediatric Mock Codes

**DOI:** 10.1093/jbcr/irad045.199

**Published:** 2023-05-15

**Authors:** Emily Snyder

**Affiliations:** Medical City Plano, Plano, Texas

## Abstract

**Introduction:**

Pediatric mock codes (PMC) were first introduced to the Burn Trauma ICU (BTICU) in Fall 2020. The goal of the PMCs were to increase efficiency in a code situation and to increase nursing confidence. However, due to the unique timing of initiation, this hospital also chose to look at how turnover rates affected the outcomes of the PMCs. The time period chosen was during the COVID crisis and the unit had an increasing number of nurses leaving for contract positions.

**Methods:**

The PMCs created were specifically designed to replicate the pediatric population the team cares for. A different PMC scenario was presented every six months for a total of four mock codes. The nurses completed the PMCs with a team approach, with each team consisting of two to six nurses. During the mock code, the nurse educator kept track of time, monitored role assignments and completion of major tasks, and ensured pre and post surveys were completed. The pre and post surveys evaluated the nurse’s self-confidence, biggest concern regarding a pediatric code, and if they had previously participated in a PMC.

**Results:**

Over the period of two years, 82 participants were captured. However, these were not unique individuals, as some nurses participated in all four mock codes. It was expected that each time the mock code was completed, the number of nurses who had participated in PMC would increase. However, as time progressed, the rate of previous participation actually decreased.

Instead of improving on time to compressions or medication administration time, the times stayed the same over the two-year time frame. When evaluating the comments on the survey, the staff concerns also did not change over time. Medication administration remained the number one concern across all four mock codes. Even though the PMC did not improve skills, 100% of those who participated expressed an increase in confidence level.

**Conclusions:**

Nursing units depend on novice nurses gaining knowledge and skills and progressing into the expert nurses on the unit. However, the loss of permanent staff and the hiring of new nurses has had an impact on this cycle. The unit lost many of its expert nurses before the new nurses had time to advance. This can be directly seen in the turnover rate and the previous participation in the PMC. Essentially this halted the unit as a whole from growing their skill set in the pediatric population. However, as the nursing market stabilizes, there are future plans to continue to evaluate the PMC skills and outcomes.

**Applicability of Research to Practice:**

As nursing turnover rates increase, patient complications and outcomes should also be evaluated to see if a correlation is present.

